# Cholesterol-conjugated *let-7a* mimics: antitumor efficacy on hepatocellular carcinoma in vitro and in a preclinical orthotopic xenograft model of systemic therapy

**DOI:** 10.1186/1471-2407-14-889

**Published:** 2014-11-28

**Authors:** Yang Ming Liu, Yu Xia, Wei Dai, Hua Ye Han, Yu Xue Dong, Jiong Cai, Xuan Zeng, Feng Yu Luo, Tao Yang, Yuan Zhi Li, Jie Chen, Jian Guan

**Affiliations:** Department of Pathology, Peking Union Medical College (PUMC) Hospital, PUMC & Chinese Academy of Medical Sciences (CAMS), Beijing, China; Department of Ultrasound, Peking Union Medical College (PUMC) Hospital, PUMC & Chinese Academy of Medical Sciences (CAMS), Beijing, China; The Core Laboratories Center, Institute of Basic Medical Sciences, PUMC & CAMS, Beijing, China; National Laboratory of Medical Biology, Institute of Basic Medical Sciences, PUMC & CAMS, Beijing, China; Department of Nuclear Medicine, Peking Union Medical College (PUMC) Hospital, PUMC & Chinese Academy of Medical Sciences (CAMS), Beijing, China; Department of Scientific Research, Peking Union Medical College (PUMC) Hospital, PUMC & Chinese Academy of Medical Sciences (CAMS), Beijing, China

## Abstract

**Background:**

A major challenge to the clinical utility of *let-7* for hepatocellular carcinoma (HCC) therapy is the lack of an effective carrier to target tumours. We confirmed the high transfection efficiency of cholesterol-conjugated *let-7a* miRNA mimics (*Chol*-*let-7a*) in human HCC cells, as well as their high affinity for liver tissue in nude mice. However, their antitumor efficacy via systemic delivery remains unknown.

**Methods:**

We explored the effects of *Chol-let-7a* on HCC in vitro and in vivo. Cell viability and mobility, *let-7a* abundance and the target *ras* genes was measured. Live-cell image and cell ultrastructure was observed. Antitumor efficacy in vivo was analyzed by ultrasonography, hispatholgogy and transmission electronic microscopy in a preclinical model of HCC orthotopic xenografts with systemic therapy.

**Results:**

*Chol*-*let-7a* inhibited the viability and mobility of HCC cells. *Chol-let-7a* was primarily observed in the cytoplasm and induced organelle changes, including autophagy*.* Mild changes were observed in the cells treated with negative control miRNA. *Chol-let-7a* reached HCC orthotopic tumours, significantly inhibited tumour growth, and prevented local invasion and metastasis. Compared to control tumours, *Chol-let-7a*-treated tumours showed more necrosis. Tumour cells showed no significant atypia, and mitoses were very rare after systemic *Chol-let-7a* therapy. Furthermore, *let-7a* abundance in orthotopic xenografts was coincident with a reduction in the expression of 3 human *ras* mRNAs and RAS proteins.

**Conclusions:**

*Chol-let-7a* exerted significant antitumor effects by down-regulating all human *ras* genes at the transcriptional and translational levels. *Chol-let-7a* inhibited cell proliferation, growth, and metastasis, and mainly functioned in the cytoplasm. *Chol-let-7a* represents a potential useful modified molecule for systemic HCC therapy.

**Electronic supplementary material:**

The online version of this article (doi:10.1186/1471-2407-14-889) contains supplementary material, which is available to authorized users.

## Background

Hepatocellular carcinoma (HCC) is the fifth most common cancer worldwide and the third most common cause of cancer mortality and has high recurrence rates after surgery. Chemotherapy and radiotherapy for HCC show limited efficacy and serious toxicity [1, 2]. New therapeutic strategies are urgently needed, particularly for the treatment of advanced tumours.

MicroRNAs (miRNAs) are endogenous non-coding small RNAs that repress gene expression at the post-transcriptional level by base pairing to the 3′-untranslated region of target messenger RNAs, and they have been identified as important mediators of carcinogenesis and clinical prognosis [3–6]. The most recent findings regarding the role of miRNAs in HCC confirmed that they hold promise as new tools for diagnosis and therapy [7–11]. A recent study in *C. elegans* reports that the *let-7* family negatively regulates *let-60*/RAS, and also that the *let-60*/RAS 3′-UTRs, including the 3′-UTRs of the human *ras* genes, contain multiple *let-7* complementary sites (LCSs), which allow *let-7* to regulate RAS protein expression [12]. Furthermore, *let-7* has been reported to inhibit tumour growth by down-regulating KRAS in some cancers, such as pancreatic carcinoma and lung cancer [13, 14]. Analysis with a computational screen showed that the human *n-ras*, *k-ras*, and *h-ras* mRNA 3′-UTRs have 9, 8, and 3 potential LCSs, respectively [12]. Although *ras* proto-oncogenes produced by mutations in codons 12, 13, and 61 do not play major roles in hepatocellular carcinogenesis [15], abnormal activation of the RAS pathway occurs in human HCC, and activated (GTP-bound) Pan-RAS, HRAS, KRAS, and NRAS are significantly up-regulated in human hepatocarcinogenesis [16, 17]. Thus, we hypothesize that modulation of *let-7* expression and its target RAS is a promising strategy for HCC treatment, because *let-7* might suppress HCC tumour growth by down-regulating all human *ras* genes.

Recently, antitumor effects of synthetic miRNA mimics were confirmed in vitro and in vivo [18–20]. Hou et al. showed that intratumoural administration of cholesterol-conjugated *PAK4* siRNA suppressed subcutaneous tumour growth in the SMMC-LTNM model [21]. Trang and colleagues [18] found that synthetic *miR-34a* and *let-7* mimics caused lung tumour reduction in mice. However, these mimics did not produced high miRNA levels in the liver tissues. We confirmed the significantly higher transfection efficiency of cholesterol-conjugated *let-7a* miRNA mimics (*Chol-let-7a*) in human HCC cells in vitro. Given the observed high affinity of *Chol-let-7a* for liver tissue in nude mice, we hypothesize that *Chol-let-7a* may be an ideal modified molecule for systemic HCC therapy.

In this study, we explored the effects of *Chol-let-7a* on HCC tumour cells in vitro, as well as its antitumor efficacy in an in vivo preclinical model of HCC orthotopic xenografts, to evaluate its potential as a systemically administered drug in the treatment of HCC. In addition, we explored the effects of *Chol-let-7a* on *ras* gene expression at the transcriptional and translational levels.

## Methods

### Materials and methods

#### Cell culture and mice

HepG2 and SMMC7721 cells were cultured in DMEM (Invitrogen, Carlsbad, CA, USA) supplemented with 10% foetal bovine serum (Invitrogen) and pen/strep (100 μg/mL). BALB/c nude (nu/nu) mice (6–7 weeks old, 20 ± 3 g) were purchased from the National Institutes for Food and Drug Control (lot number: 11400500001092; Beijing, China).

### MTT cell proliferation assays

Cholesterol-conjugated *let-7a* mimics (*Chol-let-7a*) and the negative control miRNA (*Chol-miRCtrl*) were purchased from Ribobio (Guangzhou, China). Cells (5 × 10^3^) were cultured in 96-well flat-bottomed plates. After 24 h of cell culture, cells were transfected with 50 nM *Chol-let-7a* or *Chol-miRCtrl* according to manufacturer instructions. Cells were cultured in 100 μL DMEM containing 10% FBS and 20 μL MTS reagent powder (Promega, Madison, WI, USA). Cells were harvested and seeded on 96-well flat-bottomed plates, which were incubated at 37°C for 4 h. After incubation for 1, 2, 3, 4, or 5 days, the absorbance at 550 nM was determined for each well.

### Invasion and migration assay

Assays of invasion and migration were performed as described in previous report [22]. For invasion assays, 5 × 10^4^ cells in serum-free media were seeded into the upper chambers of a 24-well BioCoat Matrigel invasion chamber (Becton Dickinson Labware, Franklin Lakes, NJ, USA) with an 8-μm pore polycarbonate membrane coated with Matrigel. For migration assays, 5 × 10^4^ cells were seeded into the upper chambers of a 24-well BioCoat control insert (Becton Dickinson Labware, Franklin Lakes, NJ, USA) with uncoated 8-μm pores in serum-free media. Medium with 10% FBS was added to the lower chambers as a chemoattractant. After 24 h of incubation, cells remaining on the upper surface of the membrane were removed with a cotton swab and cells that invaded through the membrane filter were fixed with 100% methanol, stained by hematoxylin and eosin, and photographed by soft BioLife DP under a microscope (Olympus BX40 with a DP70 digital camera, Tokyo, Japan). The number of invading or migrating cells was manually counted per high-power field for each condition (eight fields on each membrane were randomly selected).

### Wounding assay

Cells were grown to confluence in 25 cm^2^ cell culture flasks. Artificial wound tracks were created by scraping confluent cell monolayers with a pipette tip. After removal of the detached cells by gentle washing with PBS, the cells were fed with fresh complete medium and incubated to allow cells to migrate into the open area. The ability of the cells to migrate into the wound area was assessed at 24, 48, and 72 h after scratching by comparing the wound tracks in micrographs of 3 randomly selected wound areas.

### Quantitative real-time PCR and reverse transcription PCR

Total miRNA from HCC cells or snap-frozen HepG2 xenografts was isolated using the mirVANA™ PARIS™ RNA isolation kit (Applied Biosystems, Carlsbad, CA, USA). RNA (10 ng) was reverse-transcribed with the miRNA Reverse Transcription Kit (Applied Biosystems) and *let-7a* specific primers (TaqMan miRNA assay, Applied Biosystems).

Total RNA was extracted from HCC cells or snap-frozen HepG2 xenografts using the IllustraRNA spin Mini RNA Isolation Kit (GE Healthcare UK Limited, Amersham Place, Little Chalfont, UK). cDNA was synthesized using SuperScript TM III First-Strand Synthesis SuperMix for quantitative real-time reverse transcription PCR (qRT-PCR; Invitrogen Corporation, Carlsbad, CA, USA) and primers specific for the 3 human *ras* genes (TaqMan miRNA assay, Applied Biosystems).

Quantitative PCR was performed using RNU6 or *GAPDH* as a housekeeping control with an ABI Prism 7500 Sequence Detection System (Perkin-Elmer Applied Biosystems, Foster City, USA) and the Perkin-Elmer Biosystems analysis software in a manner consistent with the manufacturer’s instructions. Relative expression was calculated using the 2^-ΔΔ*C*T^method [23].

### Western blotting

HCC cells and tissues from snap-frozen HepG2 xenografts were lysed using RIPA lysis buffer (Applygen Technologies, Beijing, China). Proteins were quantified using a BCA protein kit (Applygen). Proteins (50 μg) were separated by SDS-PAGE and transferred to an Immobilon-P membrane (Millipore, Billerica, MA, USA). The membranes were blocked in 5% non-fat milk and incubated with primary antibodies. The membranes were washed in PBS-T (PBS and 0.1% Tween-20) and incubated with a peroxidase-conjugated secondary antibody (KPL, Gaithersburg, MD, USA), followed by development with a chemiluminescent substrate (Applygen). The Gel-Doc imaging system was used to scan images on Kodak film. Antibodies for KRAS, HRAS, and NRAS were purchased from Santa Cruz Biotechnology (Santa Cruz, CA, USA). GAPDH and beta-actin (β-action) antibodies were obtained from Proteintech (Chicago, IL, USA).

### Transfection, live-cell imaging, and transmission electron microscopy

HepG2 and SMMC771 cells were labelled with GFP. *Chol-let-7a* and negative control mimics labelled with Cy5 were purchased from Ribobio (Guangzhou, China).

The GFP-labelled cells (2–3 × 10^4^) were seeded in 8-well BD Falcon™ and BD BioCoat™ Culture Slides (Becton Dickinson Labware, Franklin Lakes, NJ, USA). After 48 h, cells were transfected with Cy5-labelled *Chol-let-7a* or the negative control mimics (*Chol-miRCtrl*).

For live-cell imaging, cells were continuously observed using a PerkinElmer UltraVIEW VoX-3D Live Cell Imaging System (Shanghai, China) from 24 to 72 h post-transfection. Digital images were produced using Volocity Demo software (version 5.4, 32-bit). Co-localization events were calculated using the Volocity Demo software as described in the manufacturer’s recommendations. The experiment was repeated 3 times and all samples for each individual experiment were scanned at 5 different locations.

For electron microscopy, cells were collected at 48 h and 60 h after transfection and were fixed with 2.5% glutaraldehyde for 30 min at room temperature, followed by 1.5 h in 2% OsO_4_. Samples were stained and examined with a transmission electron microscope (JEOL JEM 1010, Tokyo, Japan), and digital images were obtained with an Erlangshen ES1000W camera (Model 785, Gatan, Warrendale, PA, USA).

### In vivo experiments

All procedures were performed in accordance with the Guide for the Care and Use of Laboratory Animals (NIH publication nos. 80–23, revised 1996) and with the experimental animal welfare ethics regulations of China, with the approval of the Institution Animal Care and Use Committee of Peking Union Medical College Hospital. All animal experiments were performed at the Centre for Experimental Animal Research (CEAR), Institute of Basic Medical Sciences (IBMS), CAMS & PUMC.

### Orthotopic xenograft model with nude mice and systemic therapy with *Chol-let-7a*

HepG2 cells (2 × 10^6^) were injected directly into the livers of 20 nude mice. One week later, 18 mice with successfully engrafted HepG2 orthotopic xenografts were randomized into 3 groups of 6 animals each and examined by ultrasonography in a double-blinded manner (VisualSonics, Inc., Toronto, Ontario, Canada).

Two cohorts were treated with 5 nmol of *Chol-let-7a* or the negative control mimic (*Chol-miRCtrl*) in 250 μL saline buffer (Ribobio, Guangzhou, China) as suggested by the instruction manual. Another cohort was treated with saline buffer alone (*blank*). Systemic therapy was administered via the tail vein every 3 days for 6 weeks. Orthotopic tumour size in the liver and potential secondary metastases in the spleen were confirmed by ultrasonography with a Vevo 2100 high-frequency ultrasound system (VisualSonics, Inc., Toronto, Ontario, Canada) with measurements in 3 orthogonal axes (*a*, *b*, and *c*). Tumour volumes were determined as *V* = (*abc*)/2 [24]. The presence of tumours was confirmed via 2-dimensional vertical interfaces. Whole-animal imaging was recorded using a Kodak FX Pro in vivo imaging system. The xenograft growth curves of the 3 groups were based on the mean volume of 6 samples weekly, and inhibition was calculated based on the volume 5 weeks (the tumours of the 2 control groups at week 6 were too large for ultrasonography) after treatment as follows:


At the culmination of therapy, tumour tissues were harvested and preserved in 10% neutral buffered formalin for pathology observation. Fresh tumour tissues were snap-frozen for qRT-PCR, western blotting, or transmission electron microscopy.

### Statistical analysis

Data are expressed as the mean ± SEM. All data analyses were performed with SPSS 16.0 software (IBM, Inc., Armonk, NY, USA). Analysis of variance (ANOVA) and Student’s t-test were used for statistical comparisons between groups. *p* <0.05 was considered to be statistically significant.

## Results

### *Chol-let-7a*reduced HCC cell growth and viability in vitro

**Figure 1:**
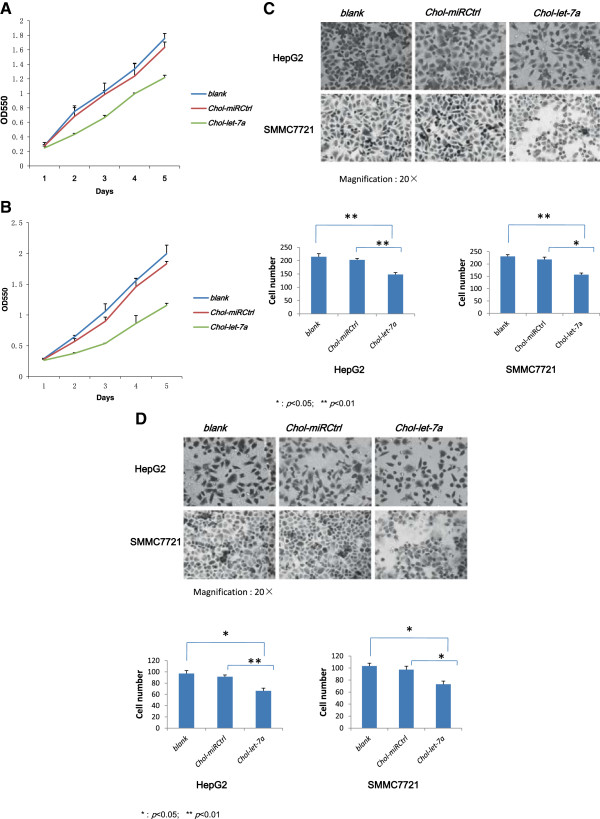
***Chol-let-7a*** **inhibited HCC cell growth and cell viability in vitro.** **A**,**B**: MTT assays show the effects of *Chol-let-7a* and *Chol-miRCtrl* on the growth of HepG2 and SMMC7721 cells. The figure shows the growth curve of tumour cells from days 1–5. *blank*, parental cells; *Chol-miRCtrl*, *Chol-miRCtrl*-transfected cells; *Chol-let-7a, Chol-let-7a-*transfected cells. **C**: A chamber-based cell migration assay showing that *Chol-let-7a* inhibits the migration of HCC cells. HepG2: *Chol-let-7a* vs. *Chol-miRCtrl* vs. *blank*, 148.3 ± 7.02 vs. 203.0 ± 5.29 vs. 214.67 ± 11.67; T-test: *Chol-let-7a* vs. *Chol-miRCtrl*, *p* = 0.001; *Chol-let-7a* vs. *blank, p* = 0.005*; Chol-miRCtrl* vs. *blank, p =* 0.207. SMMC7721: *Chol-let-7a* vs. *Chol-miRCtrl* vs. *blank*, 155.67 ± 6.66 vs. 218.33 ± 9.45 vs.230.67 ± 7.02; T-test: *Chol-let-7a* vs. *Chol-miRCtrl*, *p* = 0.021; *Chol-let-7a* vs. *blank, p* = 0.01*; Chol-miRCtrl* vs. *blank*, *p =* 0.066. Magnification: 20× **D**: The inhibitory effect of *Chol-let-7a* on cell invasion ability in the Boyden chamber invasion assay. HepG2: *Chol-let-7a* vs. *Chol-miRCtrl* vs. *blank*, 66.33 ± 4.73vs. 91.33 ± 3.21 vs. 97.00 ± 5.29; T-test: *Chol-let-7a* vs. *Chol-miRCtrl*, *p* = 0.005; *Chol-let-7a* vs. *blank*, *p* = 0.026; *Chol-miRCtrl* vs. *blank*, *p* = 0.362. SMMC7721 cells: *Chol-let-7a* vs. *Chol-miRCtrl* vs. *blank*: 73.00 ± 5.29 vs. 91.33 ± 3.21 vs.103.33 ± 4.73*.* T-test: *Chol-let-7a* vs. *Chol-miRCtrl*, *p* = 0.019; *Chol-let-7a* vs. *blank*, *p* =0.016; *Chol-miRCtrl* vs. *blank*, *p* = 0.408. Magnification: 20×.

The growth curves of HepG2 and SMMC7721 cells during the MTT assay are shown in Figure 1A and B, respectively. After 5 days, *Chol-let-7a* decreased HepG2 and SMMC7721 viability by 37.7% and 36.6%, respectively, in comparison with the parental cells (*blank*) (*p* <0.05). No significant differences in growth were observed among the 2 control HCC cell lines, the negative control miRNA mimic *(Chol-miRCtrl*)-treated cells, and the parental cells (*p* >0.05). These results verified that *Chol-let-7a* inhibited HCC cell growth in vitro.

### *Chol-let-7a*inhibited the migration and invasion of HCC cells in vitro

We determined the effects of *Chol-let-7a* on HCC cell migration and invasion, which are 2 key steps in tumour metastasis. HepG2 and SMMC7721 cells were transfected with *Chol-let-7a* or with *Chol-miRCtrl* as a negative miRNA control. The transfected *Chol-let-7a* and *Chol-miRCtrl* cells and the parental cells were used in the migration and invasion assay 48 h post-transfection.

A chamber-based cell migration assay revealed that the number of *Chol-let-7a*-treated HCC cells that migrated through the membrane was significantly lower than the number of *Chol-miRCtrl*-treated cells (*p* <0.05) or parental cells (*blank*) (*p* <0.05) that migrated through the membrane (HepG2, 148.3 ± 7.02 (*Chol-let-7a*) vs. 203.0 ± 5.29 (*Chol-miRCtrl*) vs. 214.67 ± 11.67 (*blank*); SMMC7721, 155.67 ± 6.66 (*Chol-let-7a*) vs*.* 218.33 ± 9.45 (*Chol-miRCtrl*) vs*.* 230.67 ± 7.02 (*blank*)) (Figure 1C). There were no significant differences between the 2 control groups (*p >*0.05), indicating that *Chol-let-7a* suppressed HCC cell migration.

We also used an in vitro wound healing assay to measure cell migration (data not shown). Healing speed was slower and gaps were wider in the *Chol-let-7a*-treated HepG2 and SMMC7721 cells at each time point (24, 48 h, and 72 h) in comparison with their respective control groups. At 48 h, most gaps in the 2 control cell groups were completely closed; whereas the gaps in the *Chol-let-7a*-treated cells remained open. Consistent with the results from the chamber-based cell migration assay, these data indicated that *Chol-let-7a* inhibited HCC cell migration.

Next, we evaluated the ability of HCC cells to pass through the extracellular matrix (ECM) in a Boyden chamber invasion assay. We found significantly fewer invading cells in the *Chol-let-7a-*treated group in comparison with the 2 control groups (Figure 1D) (66.33 ± 4.73 (*Chol-let-7a*) vs. 91.33 ± 3.21 (*Chol-miRCtrl*) vs. 97.00 ± 5.29 (*blank*); *Chol-let-7a* vs. the 2 control groups, both *p* <0.05), and similar results were observed with SMMC7721 cells (73.00 ± 5.29 (*Chol-let-7a*) vs. 91.33 ± 3.21 (*Chol-miRCtrl*) vs. 103.33 ± 4.73 (*blank*); *Chol-let-7a* vs. the 2 control groups, both *p* <0.05)*.* Thus, it appears that *Chol-let-7a* affects cell migration and invasion.

### Up-regulated *let-7a*down-regulated *ras*/RAS expression in HCC cells

We measured *let-7a* levels by quantitative real-time PCR 48 h after transfection with *Chol-let-7a* and *Chol-miRCtrl*. Using miRNA-specific primers, *let-7a* up-regulation in comparison with parental HCC cells and *Chol-miRCtrl*-treated control cells was confirmed in *Chol-let-7a*-treated cells (see Additional file 1A). Next, we analysed the expression of *let-7* target *ras* genes at the transcriptional and translational levels 48 h after transfection. Western blotting revealed a marked decrease in KRAS, HRAS, and NRAS protein abundance in the *Chol-let-7a-*treated HepG2 and SMMC7721 cells (see Additional file 1B). Quantitative real-time PCR (qRT-PCR) was used to measure *k-ras, h-ras*, and *n-ras* transcript abundance in the *Chol-let-7a*-treated HCC cells, and these 3 *ras* genes were found to be reduced by *Chol-let-7a* treatment (see Additional file 1C). These results verified our hypothesis that *Chol-let-7a* would inhibit the transcription and translation of all 3 human *ra*s genes in vitro.

### Live-cell images

**Figure 2:**
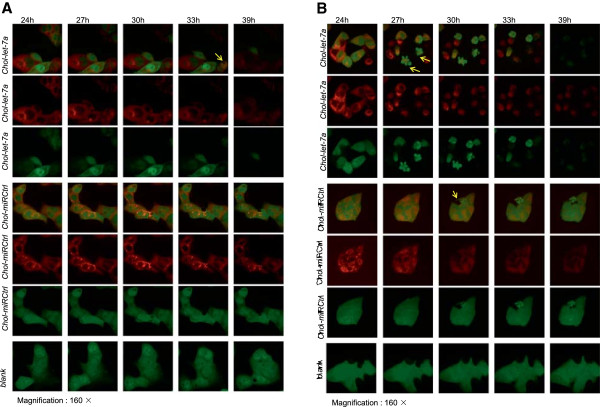
**Live-cell images.** Live-cell images were taken from 24 h to 39 h post-transfection. The parental cells with no treatment were observed as blank controls. Laser confocal images of GFP-labelled live cells (green) were recorded in the 3 groups. Cy5-labelled *Chol-let-7a* or *Chol-miRCtrl* appears as distinct red bodies. GFP-labelled living HepG2 or SMMC7721 cells appear green. Dead cells lose their green fluorescence. The images show more dead or apoptotic cells in *Chol-let-7a*-treated cells (yellow arrows). **A**: HepG2 cells. **B**: SMMC7721 cells. Magnification: 160×.

Images of live HCC cells were taken after treatment with *Chol-let-7a* or the negative control miRNA (*Chol-miRCtrl*). The parental cells served as blank controls that were visually inspected to evaluate potential off-target interactions of *Chol-miRCtrl*. Living HepG2 and SMMC7721 cells labelled by GFP were identified by green fluorescence. Images taken at the various observation time points are shown in Figure 2. The red fluorescence that indicated *Chol-let-7a* and *Chol-miRCtrl* was primarily focused in the cytoplasm (Figure 2). Through analysis of live images, we found that most of the *Chol-let-7a*-treated cells lost GFP fluorescence earlier than the 2 control groups (Figure 2). Some *Chol-let-7a*-treated cells showed typical features of apoptosis (Figure 2, Additional file 2). The numbers of GFP positive cells in the *Chol-let-7a*, *Chol-miRCtrl*, and parental cell groups were 40/50, 49/50, and 49/50, respectively, at 24 h after transfection, and 5/50, 40/50, and 49/50, respectively, at 39 h after transfection. Approximately 10/50 cells had lost GFP fluorescence in the *Chol-miRCtrl* group at 39 h after transfection. In addition, a few cells in which cytoplasmic *Chol-let-7a* was observed did not undergo cell death, and these cells subsequently lost their red fluorescence. This observation shows that some *Chol-let-7a*-treated cells did not die.

Continuous observation of the images showed that cell proliferation and mobility decreased in the *Chol-let-7a-* and *Chol-miRCtrl-*treated cells. In addition, images revealed that cell viability differed between the 2 control groups (Figure 2). There were no significant differences in proliferation and cell mobility between the 2 control groups immediately after *Chol-miRCtrl* transfection. However, the *Chol-miRCtrl-*treated cells exhibited poorer survival than the parental tumour cells (*blank*) from 39 h after transfection, and few living cells with GFP fluorescence were observed 72 h after treatment, whereas the parental cells were still active with respect to proliferation, growth, and mobility at this time point.

### Ultrastructure features

**Figure 3:**
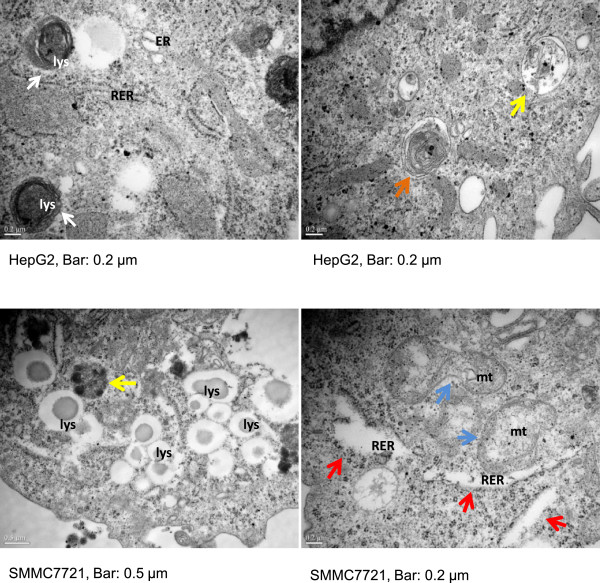
**Organelle changes after** ***Chol-let-7a*** **therapy under transmission electron microscopy.** HepG2 and SMMC7721 cells were transfected with *Chol-let-7a* or the negative control miRNA mimic (*Chol-miRCtrl*). Cells were collected at 48 h and 60 h and observed under TEM. The figure shows the cytoplasmic ultrastructure of *Chol-let-7a*-treated cells at 48 h post-transfection. Also shown are heterolysosomes as phagophores (white arrows), multivesicular bodies (MVBs, yellow arrows), and multilamellar bodies (MLBs, orange arrows). Mitophagosomes (double-membrane-enclosed damaged mitochondria) and enlarged irregular mitochondria (mt) are shown with blue arrows. Red arrows indicate the dilated RER with degranulation. Scale bars are 0.5 μm or 0.2 μm.

We observed *Chol-let-7a*- and *Chol-miRCtrl-*treated cells under transmission electronic microscopy (TEM) at 48 h and 60 h post-transfection. Abnormal organelles were observed in the cytoplasm of *Chol-let-7a*-treated cells (Figure 3). Increased autophagocytic activity in HepG2 and SMMC7721 cells was observed 48 h after *Chol-let-7a* treatment, as revealed by the presence of abundant lysosomes and phagolysosomes exhibiting heterolysosomes such as phagophores, multivesicular bodies (MVBs), and multilamellar bodies (MLBs) in the cytoplasm (Figure 3), but only slight changes in nuclear morphology were observed. Enlarged irregular mitochondria with disorganized mitochondrial crests and dilated rough endoplasmic reticulum (RER), which are often accompanied by degranulation, were also clearly observed in the *Chol-let-7a*-treated cells. Furthermore, vacuolated organelles were found in individual cells. Some changes observed in the *Chol-let-7a-*treated cells were also found in *Chol-miRCtrl-*treated HCC cells (see Additional file 3A); however, these effects were relatively mild in the negative control cells.

Long-term treatment produced significant ultrastructure modifications. In the cytoplasm of *Chol-let-7a*-treated cells, mitochondria, heterolysosomes, and RER were vacuolated and showed irregular and unclear contours and structures (see Additional file 3B), and apoptotic and necrotic cells were clearly observed 60 h after treatment. In the *Chol-miRCtrl* group, a few cells underwent death. However, interestingly, cellular morphology did not show characteristics associated with apoptotic cells. Apoptotic nuclear changes, such as nuclear shrinkage and nuclear fragmentation, were barely observed in *Chol-let-7a*-treated cells. In comparison with the rapid changes in cytoplasmic organelles, nuclear damage was strikingly delayed after *Chol-let-7a*-treatment (see Additional file 3C).

### Up-regulated *let-7a*down-regulated *ras*/RAS expression after systemic delivery

**Figure 4:**
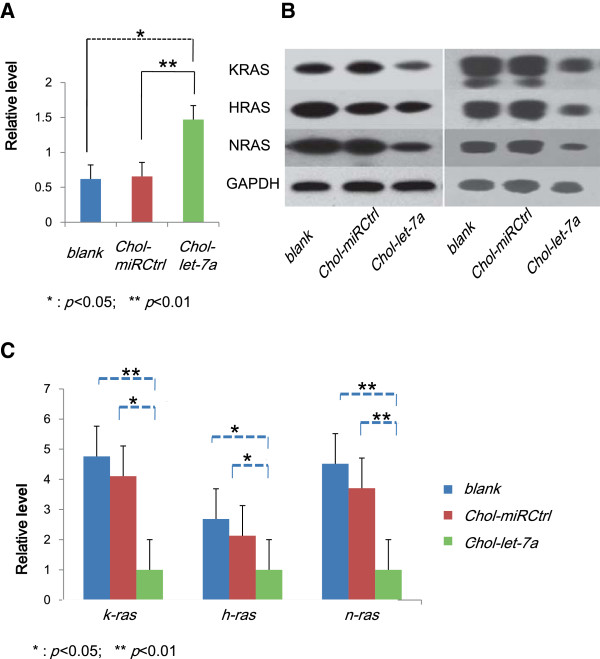
**Systemic** ***Chol-let-7a*** **therapy modulated** ***ras*** **/RAS abundance in HCC orthotopic xenografts. A**: *Let-7a* expression in HepG2 orthotopic xenografts was examined by quantitative real-time PCR. Relative quantification of *let-7a* was calculated using the comparative cycle threshold (CT) method (2 ^ΔΔct^) with *let-7a* normalized to U6. The results shown represent the mean and standard error from 3 independent experiments, *p <0.05, **p <0.01 in comparison with controls. T-test: *Chol-let-7a* vs. *Chol-miRCtrl*, *p* = 0.008; *Chol-let-7a* vs. *blank*, *p* = 0.013; *Chol-miRCtrl* vs. *blank*, *p* = 0.128. **B**: RAS protein expression in xenografts examined by western blotting. KRAS, HRAS, and NRAS protein expression was measured in xenografts by western blotting. Representative data are shown from 2 experiments. **C**: qRT-PCR analysis of *ras* mRNA in xenografts. Relative quantification of target genes was calculated using the comparative cycle threshold (CT) method (2 ^ΔΔct^) with genes normalized to *GAPDH*. The results shown represent the mean and standard error from 3 independent experiments.*p <0.05, ** *p* <0.01 in comparison with controls. Analysis revealed deregulated expression of *k-ras, h-ras*, and *n-ras* mRNA. T-test: *n-ras* (*Chol-let-7a* vs. *Chol-miRCtrl*, *p* = 0.002; *Chol-let-7a* vs. *blank*, *p* = 0.002; *Chol-miRCtrl* vs. *blank*, *p* = 0.837); *h-ras* (*Chol*-*let-7a* vs. *Chol-miRCtrl*, *p* = 0.016; *Chol-let-7a* vs. *blank*, *p* = 0.033; *Chol-miRCtrl* vs. *blank*, *p* = 0.801); and *k-ras* (*Chol-let-7a* vs. *Chol-miRCtrl*, *p* = 0.041; *Chol-let-7a* vs. *blank*, *p* = 0.005; *Chol-miRCtrl* vs. *blank*, *p* = 0.84).

To confirm that *Chol-let-7a* effectively carried *let-7a* to target tumours in vivo, we measured *let-7a* abundance in HepG2 orthotopic xenografts by qRT-PCR after systemic therapy. Using miRNA-specific primers, we found significant increases in *let-7a* miRNA abundance in treated xenografts (Figure 4A) (*Chol-let-7a* vs*. Chol-miRCtrl*, *p* = 0.008; *Chol-let-7a* vs. *blank*, *p* = 0.013; *Chol-miRCtrl* vs. *blank*, *p* =0.128). These results verified that *Chol-let-7a* successfully reached tumour tissues.

We analysed the expression of RAS proteins by western blotting and observed marked decreases in KRAS, HRAS, and NRAS abundance in *Chol-let-7a-*treated xenografts (Figure 4B). Similarly, deregulated mRNA expression of *k-ras*, *h-ras*, and *n-ras* was also investigated by qRT-PCR. The expression of *n-ras* was inhibited most significantly by *Chol-let-7a* (*p* <0.01), and expression levels of *h-ras* and *k-ras* (*p* <0.05) were also reduced (Figure 4C). These results suggest that *Chol-let-7a* successfully carried *let-7a* mimics into target HCC tumour cells and suppressed all 3 human *ras* genes at the transcriptional and translational levels, which were in accordance with our in vitro results.

### *Chol-let-7a*inhibited growth and metastasis of HCC orthotopic xenografts after systemic delivery

**Figure 5:**
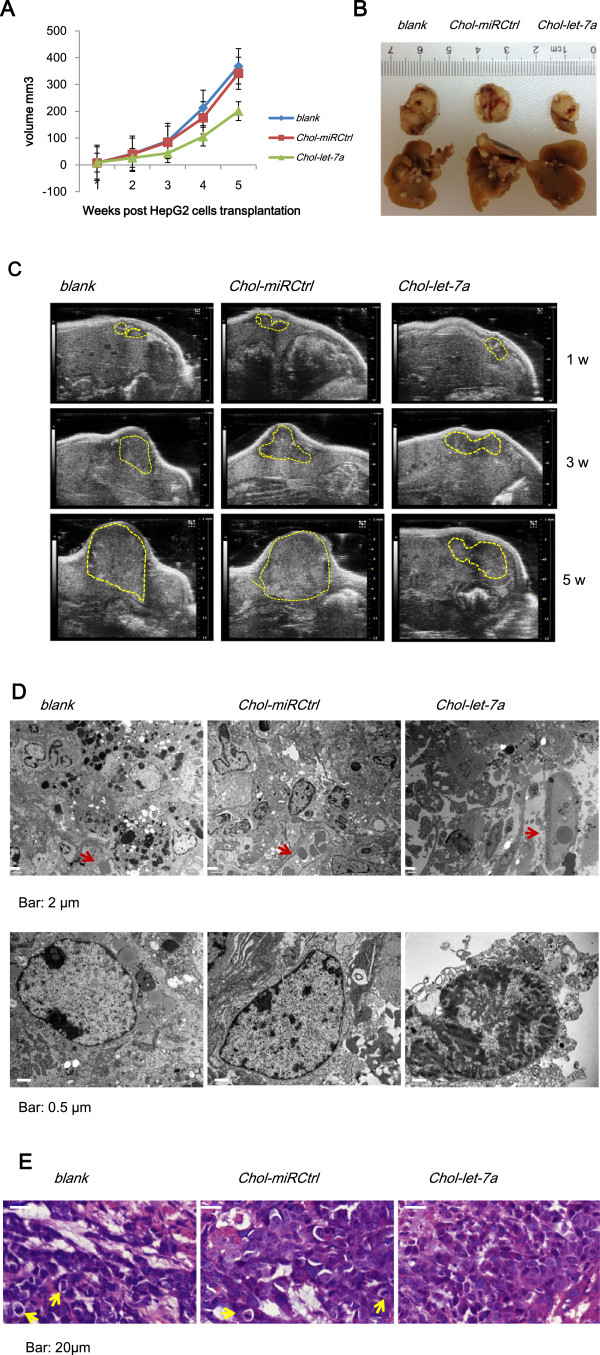
**Systemic** ***Chol-let-7a*** **therapy inhibited growth and metastasis of orthotopic HepG2 xenografts. A**: Xenograft growth curve. Representative xenografts from all groups are illustrated (N =6 mice per cohort). The *Chol-let-7a* (green), *Chol-miRCtrl* (red), and *blank* control (blue) groups are shown. **B**: Macroscopic view of the HCC orthotopic tumour and metastasis within the liver of the treatment groups. **C**: Orthotopic HepG2 xenografts examined by ultrasonography. Xenograft images were recorded 1, 3, and 5 weeks after HepG2 cells were transplanted. **D**: TEM of HCC orthotopic tumours in vivo. Necrotic and tumour cells in HCC tissues are shown. Short red arrows indicate the microvasculature in orthotopic tumours. Scale bars are 2 μm or 0.5 μm. **E**: H&E staining of orthotopic HCC tumour tissues. H&E staining was performed at the culmination of *Chol-let-7a* therapy. Differentiation and mitoses (short yellow arrows) of HCC cells of the 3 treatment groups are shown; all were recorded beside necrotic areas. Scale bars are 20 μm.

To study antitumor efficacy in vivo, we examined the size of HepG2 orthotopic xenografts of different groups by ultrasound weekly after cell transplantation. As shown in Figure 5, the growth of orthotopic tumours was significantly inhibited following *Chol-let-7a* therapy (Figure 5A-C). One week after HepG2 cell transplantation, the xenografts (Volume, mm^3^) of the *Chol-let-7a* group (8.2854 ± 2.122593) were slightly larger than those of the 2 control groups (*Chol-miRCtrl*, 7.8614 ± 1.69912; *blank*, 7.0574 ± 1.340323), while the xenografts of the *Chol-let-7a*-treated group (152.1528 ± 38.43266) were significantly smaller than those of the 2 control groups (*Chol-miRCtrl*, 424.3472 ± 60.10395; *blank*, 380.2284 ± 74.83116) at the culmination of therapy. The inhibitory rates produced by *Chol-let-7a* and *Chol-miRCtrl* on xenografts were 45.49% (*p* <0.01) and -7.13% (*p* >0.05), respectively, in comparison with the blank control group that was treated with saline buffer alone*.*

Beginning 2 weeks after *Chol-let-7a* treatment, inhibition of tumour metastasis was observed by ultrasonography. Metastases within the liver are shown in Figure 5B. Local invasion and metastasis to the spleen were inhibited in the *Chol-let-7a-*treated group (data not shown).

Under light microscopy, small and large necrosis foci were observed in tumour tissues from all 3 groups, but more necrosis was observed in the *Chol-let-7a*-treated xenografts, and necrosis was also observed in capillary-rich areas. In contrast, significant necrosis was typically observed in the central tumour area in the control groups. These necrotic features were confirmed under TEM (Figure 5D). In addition, *Chol-let-7a*-treated tumour cells showed no significant atypia, and mitoses were very rare per unit of measurement in most areas in comparison with the control groups (Figure 5E). Immunohistochemical staining for Ki-67 and ultrastructure changes in HCC cells in the orthotopic xenografts showed similar features (data not shown). These results suggest that *Chol-let-7a* inhibits tumour growth by promoting cell death and inhibiting cell proliferation.

## Discussion

We confirmed the significant antitumor efficacy of *Chol-let-7a* on HCC, and in particular its significant effect on HepG2 orthotopic xenografts after systemic delivery in a preclinical animal model. *Chol-let-7a* effectively carried *let-7a* mimics to target tumours in vivo and inhibited tumour growth, metastasis within the liver, and local invasion and metastasis to the spleen. Significant increases in *let-7a* miRNA abundance were observed in *Chol-let-7a*-treated xenografts. Moreover, *let-7a* abundance in HepG2 orthotopic xenografts was coincident with a reduction in the expression of 3 human *ras* mRNAs and RAS proteins. These results suggest that *Chol-let-7a* inhibits HCC cell growth by regulating all 3 human *ras* genes at the transcriptional and translational levels.

We found some different features in the orthotopic xenograft tissues after *Chol-let-7a* systemic therapy in comparison with the 2 control groups. All tumour tissues contained small and large necrosis foci, but more necrosis was observed in the *Chol-let-7a*-treated xenografts, and necrosis was also observed in capillary-rich areas. In contrast, significant necrosis was typically observed in the central areas of tumours in the control groups. The histopathological features of *Chol-let-7a*-treated xenografts may have been induced by the type of *Chol-let-7a* transportation used in this study. Tumour cells in capillary-rich areas could be more susceptible than other cells to systemically administered *Chol-let-7a* molecules. In addition, well-differentiated tumour cells with no significant atypia and only very rare mitoses were observed after *Chol-let-7a* therapy. These results suggest that *Chol-let-7a* inhibited tumour growth by inhibiting cell proliferation and promoting cell death.

We confirmed that *Chol-let-7a* entered cells and functioned primarily in the cytoplasm based on morphology and ultrastructure analysis. This result was consistent with the potential functional basis of *let-7a*, which involves the inhibition of target *ras* genes at the transcriptional and translational levels. In vitro, we observed the effects of *Chol-let-7a* on HCC cells by living cell image analysis and transmission electron microscopy. Both results suggested that *Chol-let-7a* entered cells and functioned primarily in the cytoplasm. The red fluorescence that indicated *Chol-let-7a* and *Chol-miRCtrl* was primarily focused in the cytoplasm. TEM revealed that *Chol-let-7a* damaged some cytoplasmic organelles, but only slight changes in nuclear morphology were observed. Cellular nuclear morphology did not show characteristics associated with apoptotic cells even at 60 h after *Chol-let-7a* therapy, when long-term treatment had produced significant ultrastructure modifications in the cytoplasm. Apoptotic nuclear changes such as shrinkage and fragmentation were barely observed in *Chol-let-7a*-treated cells, including those in which mitochondria, heterolysosomes, and RER were vacuolated and showed irregular and unclear contours and structures. In comparison with the rapid changes in cytoplasmic organelles, nuclear damage was strikingly delayed after *Chol-let-7a*-treatment. Autophagocytic activity was observed in *Chol-let-7a-* treated cells. Therefore, we suggest that autophagy may be an important mechanism through which *Chol-let-7a* produces antitumor effects [25].

We previously examined the antitumor effect of *Chol-let-7a* on HCC by using intratumoural administration in a subcutaneous xenograft model. Results showed that intratumoural administration of *Chol-let-7a* reduced tumour growth; however, cell phenotype and morphology in most areas of the subcutaneous xenografts showed no such changes, and these areas showed actively growing cells with high rates of mitosis (data not shown). Therefore, *Chol-let-7a* produces better inhibitory effects when it is systemically administered. Because of the high affinity of *Chol-let-7a* for liver tissue in nude mice (data not shown) and the convenience of systemic administration, *Chol-let-7a* represents a potential useful modified molecule for systemic HCC therapy.

However, the delivery system may have off-target effects, as indicated by the differences observed between the *Chol-miRCtrl* cells and the parental cells. Cell viability differed between the 2 control groups. The *Chol-miRCtrl-*treated cells exhibited poorer survival than the parental tumour cells from 39 h after transfection, and few living cells with GFP fluorescence were observed 72 h after treatment, whereas the parental cells were still active with respect to proliferation, growth, and mobility at this time point. This result suggests that *Chol-miRCtrl* can reduce the viability of some cells. We compared the effects of *Chol-let-7a* and *Chol-miRCtrl* on HepG2 cells at 3 different doses. In comparison with the 25 nM and 50 nM doses, 100 nM *Chol-miRCtrl* slightly slowed cell growth 72 h after transfection (see Additional file 4A), but there were no differences in cell growth between the treatment groups at 48 h (see Additional file 4B). These results suggest that dosage and prolonged action time contribute to the off-target effects of *Chol-miRCtrl*.

Ultrastructure features also differed between the 2 control groups. Some organelle changes observed in the *Chol-let-7a*-treated cells were also found in the *Chol-miRCtrl*-treated HCC cells under TEM. In addition, autophagy was observed in some *Chol-miRCtrl* treated tumour cells that under death, indicating that autophagocytic activity could also be a potential factor induces off-target effects. Given the observed high affinity of *Chol-let-7a* for liver tissue in nude mice, we hypothesize that *Chol-let-7a* may has potential off-target effects primary in liver tissue when it is administered systemically as a therapeutic molecule. In future studies, we will investigate off-target effects of *Chol-let-7a* in preclinical animal models.

## Conclusions

We confirmed the significant antitumor efficacy of *Chol-let-7a* on HCC, and in particular its significant effect on HepG2 orthotopic xenografts after systemic delivery in a preclinical animal model. *Chol-let-7a* effectively carried *let-7a* to target tumours in vivo and inhibit tumour growth by inhibiting cell proliferation and promoting cell death. In addition, *Chol-let-7a* can inhibit HCC cell growth by regulating all 3 human *ras* genes at the transcriptional and translational levels. Moreover, we confirmed *Chol-let-7a* entered cells and functioned primarily in the cytoplasm, and autophagy may be an important mechanism through which *Chol-let-7a* produces antitumor effects. Taken together, *Chol-let-7a* represents a potential useful modified molecule for systemic HCC therapy. However, further studies of *Chol-let-7a*-produced off-target effects when it is systemically administered are required.

## Electronic supplementary material

Additional file 1: **Up-regulated** ***let-7a*** **down-regulated human** ***ras*** **/RAS expression in HCC cells in vitro.** A: *Let-7a* levels measured by quantitative real-time PCR 48 h post-transfection of *Chol-let-7a* or *Chol-miRCtrl*. Significant increases in *let-7a* levels in *Chol-let-7a*-treated HepG2 and SMMC7721 cells are shown. T-test: HepG2: *Chol-let-7a* vs. *Chol-miRCtrl, p* = 0.003; *Chol-let-7a* vs. *blank*, *p* = 0.003; *Chol-miRCtrl* vs. *blank*, *p* = 0.08. SMMC7721: *Chol-let-7a* vs. *Chol-miRCtrl, p* = 0.001; *Chol-let-7a* vs. *blank*, *p* = 0.001; *Chol-miRCtrl* vs. *blank*, *p* = 0.062. The results shown represent the mean and standard error from 3 independent experiments.*p <0.05, **p <0.01 in comparison with controls. (B) Expression of RAS proteins examined by western blotting 48 h after transfection of *Chol-let-7a* or *Chol-miRCtrl*. There was a marked decrease in KRAS, HRAS, and NRA*S* protein abundance in *Chol-let-7a-*treated cells. (C) Deregulated expression of *k-ras, h-ras*, and *n-ras* mRNAs as determined by qRT-PCR 48 h after transfection of *Chol-let-7a*. T-test for *k-ras* in HepG2: *Chol-let-7a* vs. *Chol-miRCtrl, p* = 0.005; *Chol-let-7a* vs. *blank*, *p* = 0.002; *Chol-miRCtrl* vs. *blank*, *p* = 0.286. T-test for *k-ras* in SMMC7721: *Chol-let-7a* vs. *Chol-miRCtrl, p* =0.008; *Chol-let-7a* vs. *blank*, *p* = 0.007; *Chol-miRCtrl* vs. *blank*, *p* = 0.463. T-test for *h-ras* in HepG2: *Chol-let-7a* vs. *Chol-miRCtrl, p* = 0.005; *Chol-let-7a* vs. *blank*, *p* = 0.001; *Chol-miRCtrl* vs. *blank*, *p* = 0.081. T-test for *h-ras* in SMMC7721: *Chol-let-7a* vs. *Chol-miRCtrl, p* = 0.032; *Chol-let-7a* vs. *blank*, *p* = 0.023; *Chol-miRCtrl* vs. *blank*, *p* = 0.907. T-test for *n-ras* in HepG2: *Chol-let-7a* vs. *Chol-miRCtrl, p* = 0.001; *Chol-let-7a* vs. *blank*, *p* = 0.004; *Chol-miRCtrl* vs. *blank*, *p* = 0.755. T-test for *n-ras* in SMMC7721: *Chol-let-7a* vs. *Chol-miRCtrl, p* = 0.001; *Chol-let-7a* vs. *blank*, *p* = 0.002; *Chol-miRCtrl* vs. *blank*, *p* = 0.958. The results shown represent the mean and standard error from 3 independent experiments.*p <0.05, **p <0.01 in comparison with controls. (PDF 383 KB)

Additional file 2: **Live cell images showing apoptosis and distribution of** ***Chol-let-7a*** **and the negative control miRNA in HCC cells.** The HCC cells were labelled with GFP. The cholesterol-conjugated *let-7a* mimics (*Chol-let-7a*), or negative control miRNA (*Chol-miRCtrl*) were labelled with Cy5 fluorescence. Laser confocal images of GFP-labelled HepG2 and SMMC7721 cells (green) treated with 50 nM Cy5-labelled *Chol-let-7a* or *Chol-miRCtrl* are shown. The images are of HepG2 cells at 1 day after injection. Cy5 fluorescence appears in the cytoplasm as distinct red bodies surrounding the nucleus. Yellow arrow indicates an apoptotic cell in *Chol-let-7a-*treated group. (PDF 507 KB)

Additional file 3: **HCC cells after** ***Chol-let-7a*** **or** ***Chol-miRCtrl*** **treatment observed by TEM.** *blank*: Parental HCC cells; *Chol-let-7a*: *Chol-let-7a*-treated HCC cells; *Chol-miRCtrl*: *Chol-miRCtrl*-treated HCC cells A: HepG2 and SMMC7721 cells from the treatment groups at 48 h post-transfection. B: *Chol-let-7a*-treated cells at 60 h post-transfection. Vacuolated organelles with irregular and unclear contours and structures are shown.C: HCC cells observed under TEM. Parental, *Chol-let-7a*- and *Chol-miRCtrl*-treated HCC cells were observed under TEM at 48 h and 60 h after treatment. More dead and apoptotic cells were found in the *Chol-let-7a*-treated cells, but some *Chol-miRCtrl*-treated cells showed similar morphology. (PDF 2 MB)

Additional file 4: **MTT assay of HepG2 cells transfected with different doses of** ***Chol-let-7a*** **or** ***Chol-miRCtrl*** **.** HepG2 cells were transfected with 25 nM, 50 nM, or 100 nM of each treatment and the absorbance at 550 nM was determined for each well at 48 h and 72 h after transfection. A: MTT assay of HepG2 cells at 72h post-transfection with different doses of each treatment. At 72 h, inhibition increased in a dose-dependent manner in the *Chol-let-7a-*treated group. In addition, cells transfected with 100 nM *Chol-miRCtrl* (short arrow) grew more slowly than cells transfected with 25 nM and 50 nM *Chol-miRCtrl*. B: MTT assay of HepG2 cells at 48 h post-transfection with different doses of each treatment. At 48 h, HepG2 cells in the *Chol-let-7a* group were inhibited. In the *Chol-let-7a-*treated group, inhibition increased as the administered dose increased. No difference was observed between the *Chol-miRCtrl*-treated groups (long arrow) and the parental cells (*blank*). (PDF 235 KB)
