# Supplementary material for: Cholesterol-conjugated let-7a mimics: antitumor efficacy on hepatocellular carcinoma in vitro and in a preclinical orthotopic xenograft model of systemic therapy
Source: BMC Cancer. 2014 Nov 28;14:889. doi: 10.1186/1471-2407-14-889 (PMC4289300; doi:10.1186/1471-2407-14-889)

A： *Let-7a* levels measured by quantitative real-time PCR 48 h post-transfection of *Chol-let-7a* or *Chol-miRCtrl*

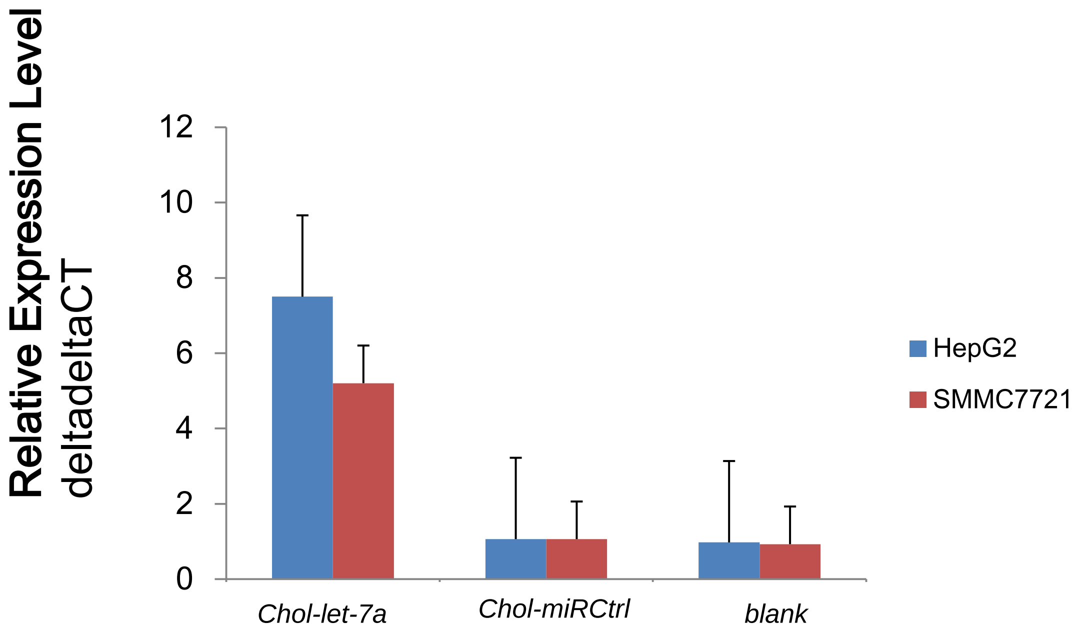

B： Expression of RAS proteins examined by western blotting 48 h post transfection

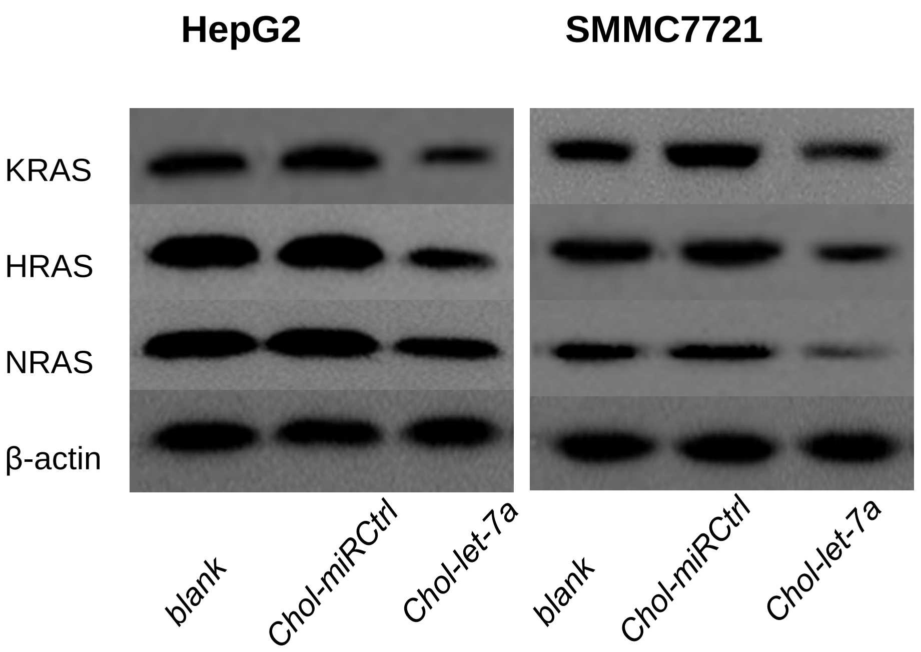

C: Deregulated expression of *k-ras, h-ras*, and *n-ras* mRNAs as determined by qRT-PCR 48 h post transfection

HepG2

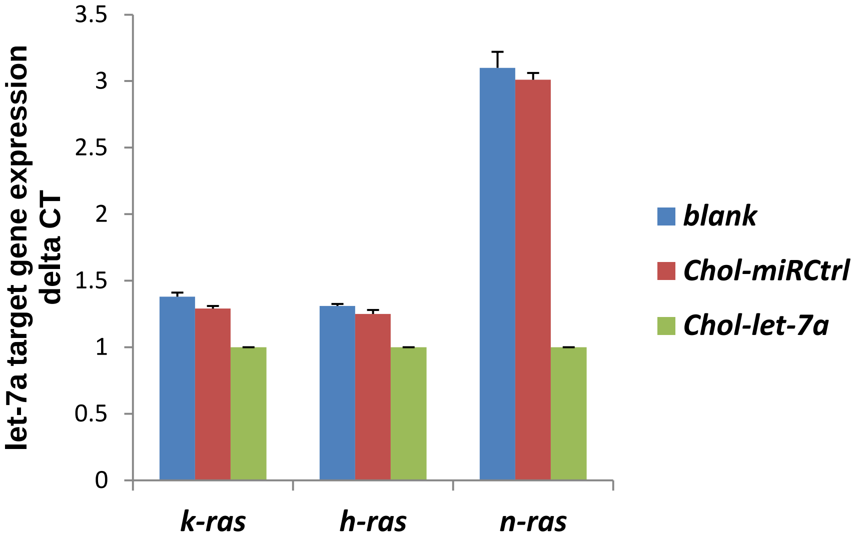

SMMC7721

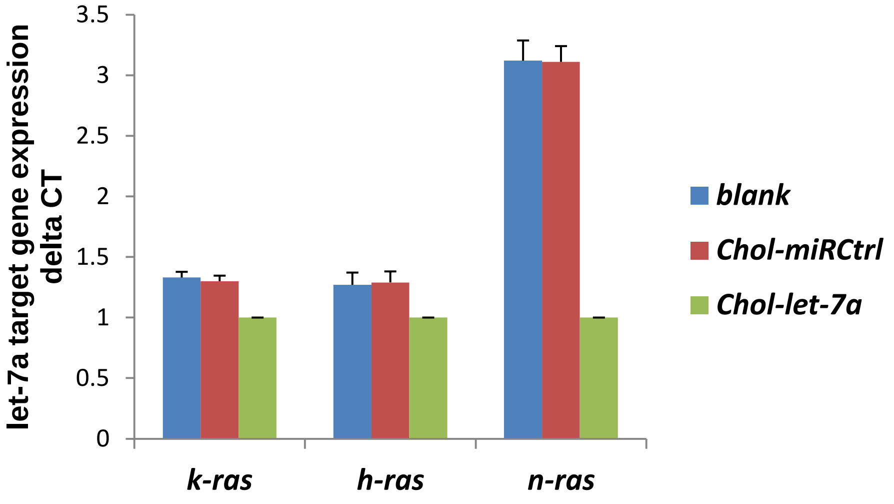

Supplement: Supplementary file 1 — Additional file 1: Up-regulated let-7a down-regulated human ras /RAS expression in HCC cells in vitro. A: Let-7a levels measured by quantitative real-time PCR 48 h post-transfection of Chol-let-7a or Chol-miRCtrl. Significant increases in let-7a levels in Chol-let-7a-treated HepG2 and SMMC7721 cells are shown. T-test: HepG2: Chol-let-7a vs. Chol-miRCtrl, p = 0.003; Chol-let-7a vs. blank, p = 0.003; Chol-miRCtrl vs. blank, p = 0.08. SMMC7721: Chol-let-7a vs. Chol-miRCtrl, p = 0.001; Chol-let-7a vs. blank, p = 0.001; Chol-miRCtrl vs. blank, p = 0.062. The results shown represent the mean and standard error from 3 independent experiments.*p <0.05, **p <0.01 in comparison with controls. (B) Expression of RAS proteins examined by western blotting 48 h after transfection of Chol-let-7a or Chol-miRCtrl. There was a marked decrease in KRAS, HRAS, and NRAS protein abundance in Chol-let-7a-treated cells. (C) Deregulated expression of k-ras, h-ras, and n-ras mRNAs as determined by qRT-PCR 48 h after transfection of Chol-let-7a. T-test for k-ras in HepG2: Chol-let-7a vs. Chol-miRCtrl, p = 0.005; Chol-let-7a vs. blank, p = 0.002; Chol-miRCtrl vs. blank, p = 0.286. T-test for k-ras in SMMC7721: Chol-let-7a vs. Chol-miRCtrl, p =0.008; Chol-let-7a vs. blank, p = 0.007; Chol-miRCtrl vs. blank, p = 0.463. T-test for h-ras in HepG2: Chol-let-7a vs. Chol-miRCtrl, p = 0.005; Chol-let-7a vs. blank, p = 0.001; Chol-miRCtrl vs. blank, p = 0.081. T-test for h-ras in SMMC7721: Chol-let-7a vs. Chol-miRCtrl, p = 0.032; Chol-let-7a vs. blank, p = 0.023; Chol-miRCtrl vs. blank, p = 0.907. T-test for n-ras in HepG2: Chol-let-7a vs. Chol-miRCtrl, p = 0.001; Chol-let-7a vs. blank, p = 0.004; Chol-miRCtrl vs. blank, p = 0.755. T-test for n-ras in SMMC7721: Chol-let-7a vs. Chol-miRCtrl, p = 0.001; Chol-let-7a vs. blank, p = 0.002; Chol-miRCtrl vs. blank, p = 0.958. The results shown represent the mean and standard error from 3 independent experiments.*p <0.05, **p <0.01 in comparison with controls. ( [file 12885_2014_5132_MOESM1_ESM.pdf]
