# Supplementary figures and images for: Cholesterol-conjugated let-7a mimics: antitumor efficacy on hepatocellular carcinoma in vitro and in a preclinical orthotopic xenograft model of systemic therapy
Source: BMC Cancer. 2014 Nov 28;14:889. doi: 10.1186/1471-2407-14-889 (PMC4289300; doi:10.1186/1471-2407-14-889)

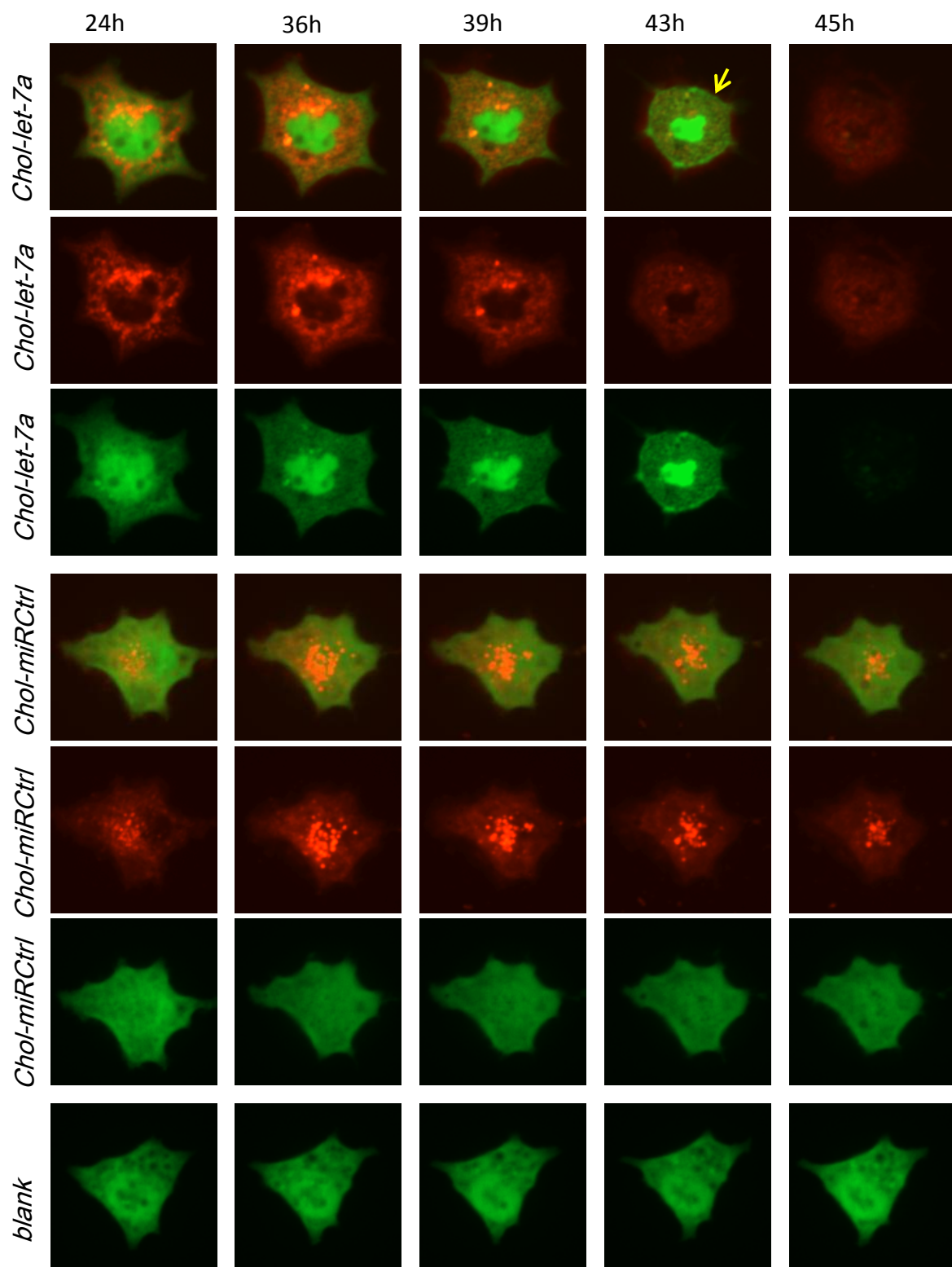

Supplement: Supplementary file 2 — Additional file 2: Live cell images showing apoptosis and distribution of Chol-let-7a and the negative control miRNA in HCC cells. The HCC cells were labelled with GFP. The cholesterol-conjugated let-7a mimics (Chol-let-7a), or negative control miRNA (Chol-miRCtrl) were labelled with Cy5 fluorescence. Laser confocal images of GFP-labelled HepG2 and SMMC7721 cells (green) treated with 50 nM Cy5-labelled Chol-let-7a or Chol-miRCtrl are shown. The images are of HepG2 cells at 1 day after injection. Cy5 fluorescence appears in the cytoplasm as distinct red bodies surrounding the nucleus. Yellow arrow indicates an apoptotic cell in Chol-let-7a-treated group. (PDF 507 KB) [file 12885_2014_5132_MOESM2_ESM.pdf]
