# Supplementary material for: Cholesterol-conjugated let-7a mimics: antitumor efficacy on hepatocellular carcinoma in vitro and in a preclinical orthotopic xenograft model of systemic therapy
Source: BMC Cancer. 2014 Nov 28;14:889. doi: 10.1186/1471-2407-14-889 (PMC4289300; doi:10.1186/1471-2407-14-889)
Supplement: Supplementary file 3 — Additional file 3: HCC cells after Chol-let-7a or Chol-miRCtrl treatment observed by TEM. blank: Parental HCC cells; Chol-let-7a: Chol-let-7a-treated HCC cells; Chol-miRCtrl: Chol-miRCtrl-treated HCC cells A: HepG2 and SMMC7721 cells from the treatment groups at 48 h post-transfection. B: Chol-let-7a-treated cells at 60 h post-transfection. Vacuolated organelles with irregular and unclear contours and structures are shown.C: HCC cells observed under TEM. Parental, Chol-let-7a- and Chol-miRCtrl-treated HCC cells were observed under TEM at 48 h and 60 h after treatment. More dead and apoptotic cells were found in the Chol-let-7a-treated cells, but some Chol-miRCtrl-treated cells showed similar morphology. (PDF 2 MB) [file 12885_2014_5132_MOESM3_ESM.pdf]

A： HepG2 and SMMC7721 cells from the treatment groups at 48 h post-transfection

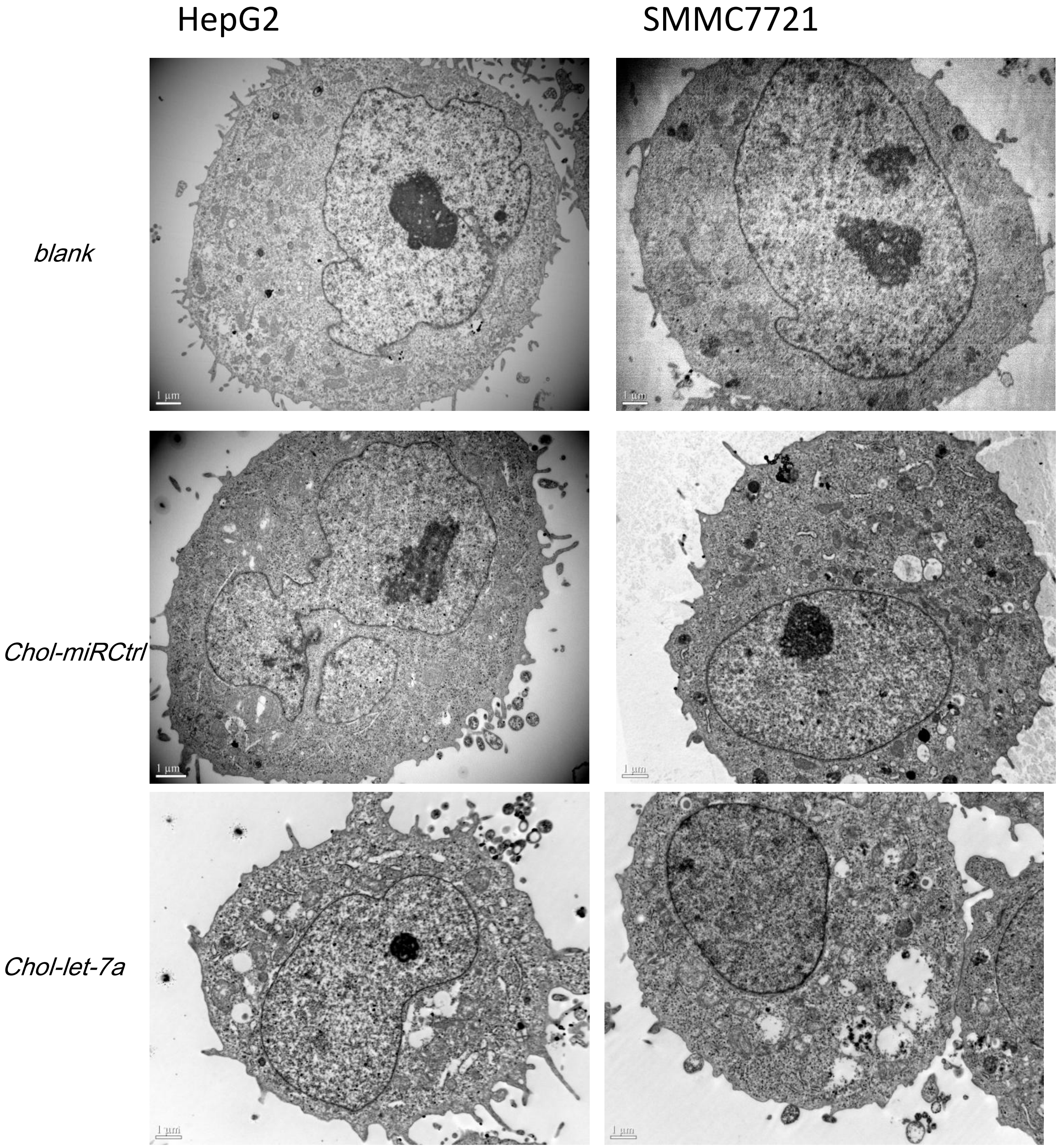

Bar: 1μm

B： *Chol-let-7a*-treated cells at 60 h post-transfection

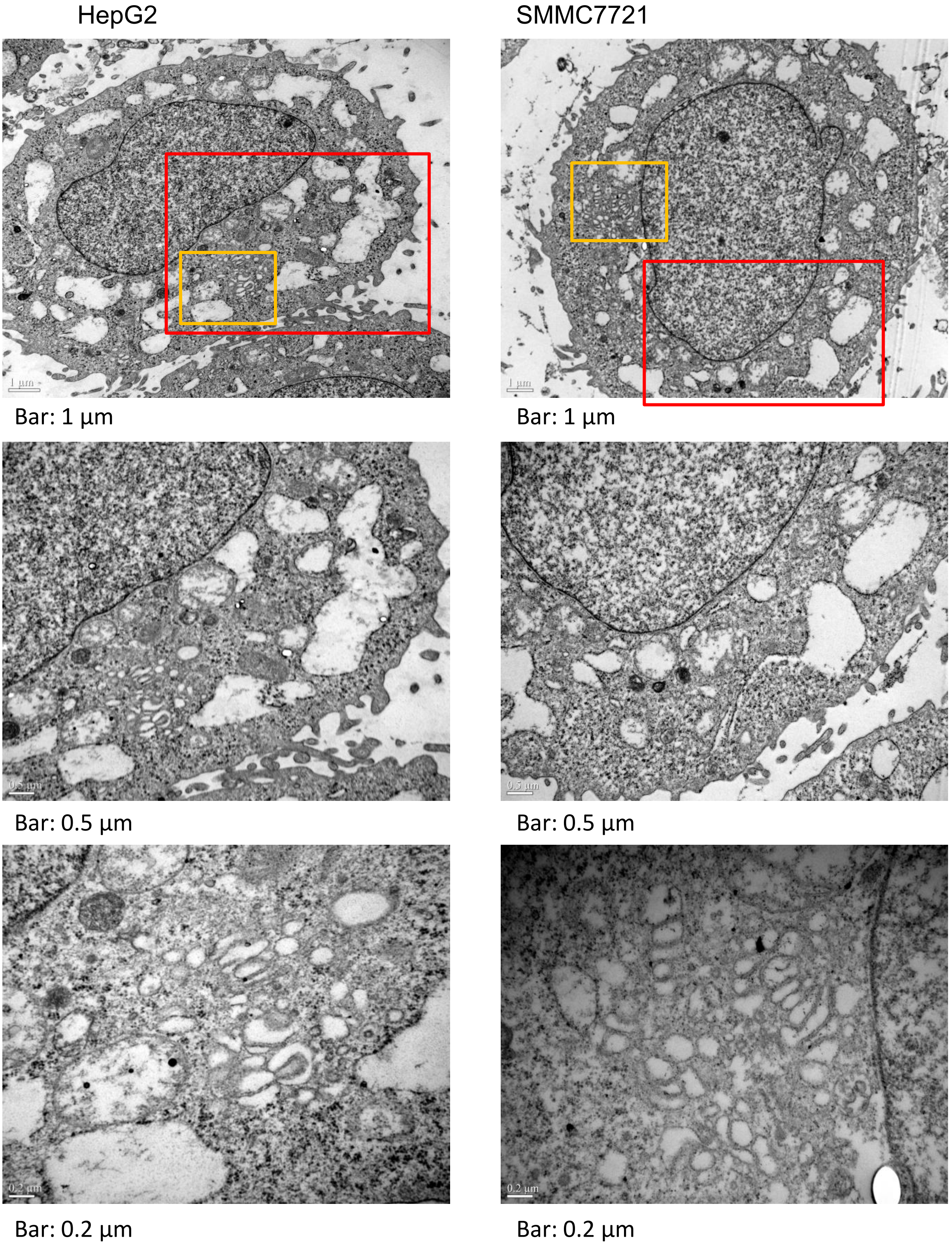

C： HCC cells observed under TEM

HepG2 **SMMC7721**

Parental cells blank

Chol-miRCtrl **48h**

Chol-Let-7a **48h**

Chol-miRCtrl **60h**

Chol-Let-7a **60h**

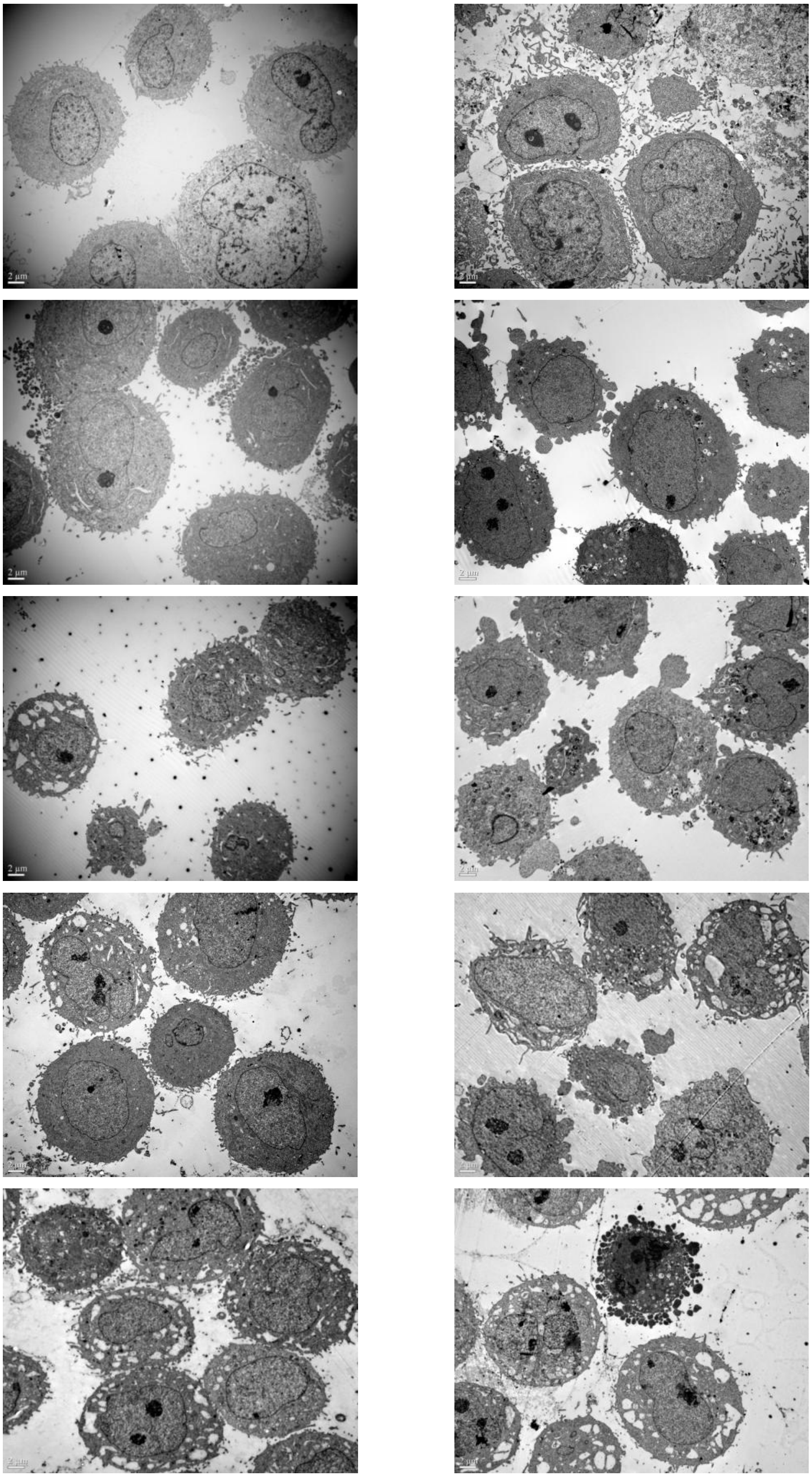

Bar: 2μm
