# Supplementary material for: Cholesterol-conjugated let-7a mimics: antitumor efficacy on hepatocellular carcinoma in vitro and in a preclinical orthotopic xenograft model of systemic therapy
Source: BMC Cancer. 2014 Nov 28;14:889. doi: 10.1186/1471-2407-14-889 (PMC4289300; doi:10.1186/1471-2407-14-889)

A： MTT assay of HepG2 cells at 72h post-transfection with different doses of each treatment

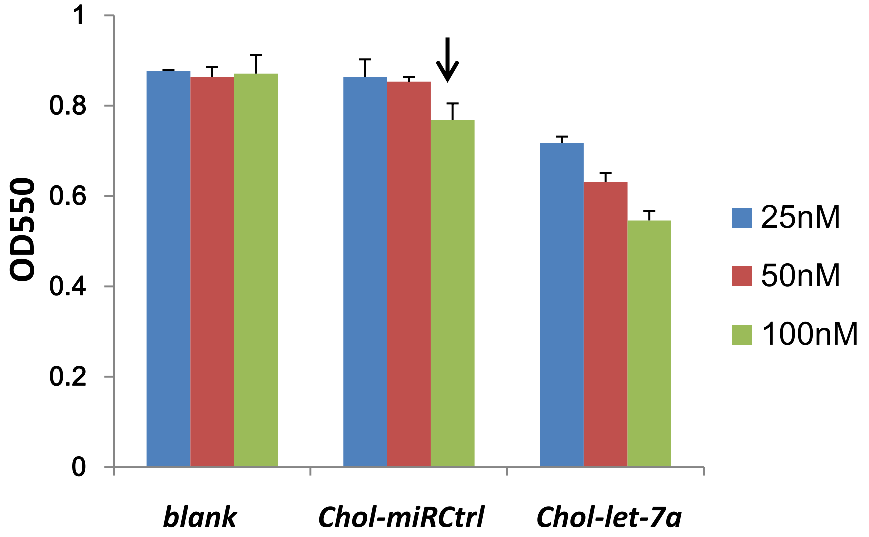

B： MTT assay of HepG2 cells at 48 h post-transfection with different doses of each treatment.

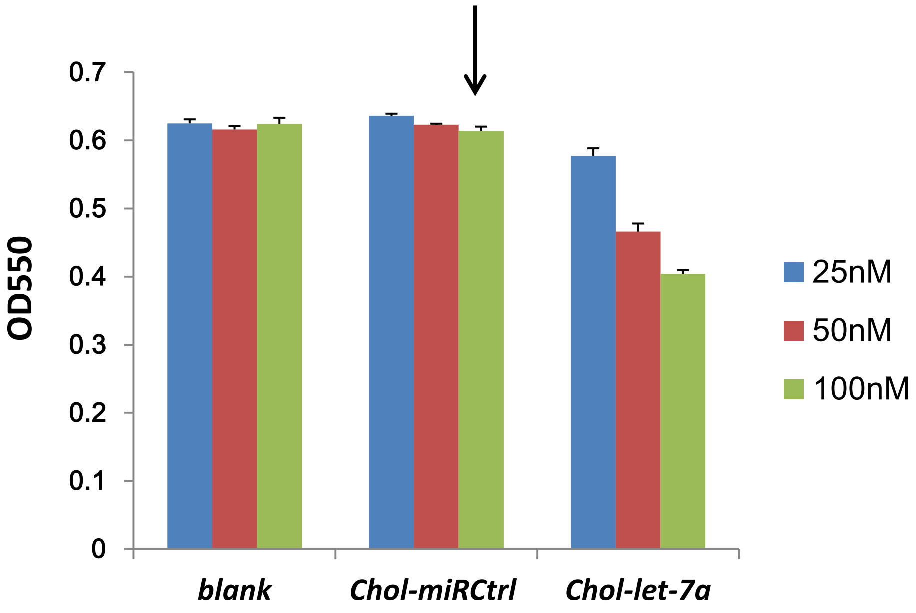

Supplement: Supplementary file 4 — Additional file 4: MTT assay of HepG2 cells transfected with different doses of Chol-let-7a or Chol-miRCtrl . HepG2 cells were transfected with 25 nM, 50 nM, or 100 nM of each treatment and the absorbance at 550 nM was determined for each well at 48 h and 72 h after transfection. A: MTT assay of HepG2 cells at 72h post-transfection with different doses of each treatment. At 72 h, inhibition increased in a dose-dependent manner in the Chol-let-7a-treated group. In addition, cells transfected with 100 nM Chol-miRCtrl (short arrow) grew more slowly than cells transfected with 25 nM and 50 nM Chol-miRCtrl. B: MTT assay of HepG2 cells at 48 h post-transfection with different doses of each treatment. At 48 h, HepG2 cells in the Chol-let-7a group were inhibited. In the Chol-let-7a-treated group, inhibition increased as the administered dose increased. No difference was observed between the Chol-miRCtrl-treated groups (long arrow) and the parental cells (blank). (PDF 235 KB) [file 12885_2014_5132_MOESM4_ESM.pdf]
